# Curcumin attenuates MSU crystal-induced inflammation by inhibiting the degradation of IκBα and blocking mitochondrial damage

**DOI:** 10.1186/s13075-019-1974-z

**Published:** 2019-08-27

**Authors:** Baofeng Chen, Hongmei Li, Guochun Ou, Long Ren, Xiaohong Yang, Mei Zeng

**Affiliations:** 1 Institute of Rheumatology and Immunology, The Affiliated Hospital of North Sichuan Medical College, 63# Wenhua Road, Nanchong, 637000 Sichuan China; 2 Sichuan Key Laboratory of Medical Imaging, North SiChuan Medical College, 234# Fujiang Road, Nanchong, 637000 Sichuan China; 3 Preclinical School of North SiChuan Medical College, 234# Fujiang Road, Nanchong, 637000 Sichuan China; 4 The Fifth People’s Hospital of Nanchong City, 21#Bajiao Street, Nanchong, 637100 Sichuan China

**Keywords:** Curcumin, NF-κB, IκBα, MSU, Gout

## Abstract

**Background:**

Gouty arthritis is characterized by the deposition of monosodium urate (MSU) within synovial joints and tissues due to increased urate concentrations. In this study, we explored the effect of the natural compound curcumin on the MSU crystal-stimulated inflammatory response.

**Methods:**

THP-1-derived macrophages and murine RAW264.7 macrophages were pretreated with curcumin for 1 h and then stimulated with MSU suspensions for 24 h. The protein level of TLR4, MyD88, and IκBα, the activation of the NF-κB signaling pathway, the expression of the NF-κB downstream inflammatory cytokines, and the activity of NLRP3 inflammasome were measured by western blotting and ELISA. THP-1 and RAW264.7 cells were loaded with MitoTracker Green to measure mitochondrial content, and MitoTracker Red to detect mitochondrial membrane potential. To measure mitochondrial reactive oxygen species (ROS) levels, cells were loaded with MitoSOX Red, which is a mitochondrial superoxide indicator. The effects of curcumin on mouse models of acute gout induced by the injection of MSU crystals into the footpad and synovial space of the ankle, paw and ankle joint swelling, lymphocyte infiltration, and MPO activity were evaluated.

**Results:**

Curcumin treatment markedly inhibited the degradation of IκBα, the activation of NF-κB signaling pathway, and the expression levels of the NF-κB downstream inflammatory genes such as IL-1β, IL-6, TNF-α, COX-2, and PGE2 in the MSU-stimulated THP-1-derived macrophages. Curcumin administration protected THP-1 and RAW264.7 cells from MSU induced mitochondrial damage through preventing mitochondrial membrane potential reduction, decreasing mitochondria ROS, and then inhibited the activity of NLRP3 inflammasome. Intraperitoneal administration of curcumin alleviated MSU crystal-induced paw and ankle joint swelling, inflammatory cell infiltration, and MPO activity in mouse models of acute gout. These results correlated with the inhibition of the degradation of IκBα, the phosphorylation levels of NF-κB subunits (p65 and p50), and the activity of NLRP3 inflammasome.

**Conclusion:**

Curcumin administration effectively alleviated MSU-induced inflammation by suppressing the degradation of IκBα, the activation NF-κB signaling pathway, the damage of mitochondria, and the activity of NLRP3 inflammasome. Our results provide a new strategy in which curcumin therapy may be helpful in the prevention of acute episodes of gout.

## Background

Gout is a common type of inflammatory arthritis in men and is triggered by the deposition of monosodium urate (MSU) crystals in articular and periarticular tissues [1]. Colchicine and NSAIDs are commonly used to treat acute gout arthritis, and these agents are only modestly effective. Furthermore, their long-term use is limited by the inevitable side effects of gastrointestinal bleeding, gastrointestinal toxicity, and nephrotoxicity as well as therapeutic gaps [2]. Therefore, researchers are increasingly interested in the active components of natural herbal plants due to their extensive range of sources, high curative effect, and fewer side effects.

Turmeric has a long history in ayurvedic medicine for treating inflammation. The main active ingredient of turmeric is curcumin [3]. In vitro and in vivo experiments have indicated that curcumin can suppress inflammation. This product has no related toxicity and plays a beneficial role in many types of inflammatory diseases, including obesity, diabetes, cardiovascular disease, bronchial asthma, and rheumatoid arthritis [4]. Many studies have shown that toll-like receptor (TLR)/ myeloid differentiation factor 88 (MyD88)/nuclear factor NF-κB, and NLR family pyrin domain containing 3 (NLRP3) signaling pathways are implicated in MSU crystal-induced inflammatory cytokine release in monocytes/macrophages [5–7]. The overexpression of COX-2 in articular tissue is characteristic of crystalline arthritis [8]. Previous studies have shown that MSU crystals can stimulate COX-2 expression in monocytes, synovial fluid inflammatory cells, rabbit synovial cell lines, human articular chondrocytes, and synovial cells [9, 10].

Previously, curcumin was shown to potentially prevent IκBα degradation by impeding 26S proteasome activity, the NF-κB signaling pathway, and NLRP3 inflammasome [11–18]. As the NLRP3 inflammasome is a key element in the MSU crystalloid-induced inflammatory response, strategies that block its activation or affect its activity could reduce gout inflammation [19]. Mitochondrial damage is closely related to the activation of NLRP3 inflammasome and the inflammatory response of gout, especially mitochondrial reactive oxygen species (ROS) which affect the assembly of NLRP3 inflammasome [20–23]. However, the effect of curcumin on MSU crystal-induced inflammation is controversial.

In this study, we explored the effects of curcumin on gout models in vitro and in vivo by assessing inflammatory cytokines, detecting paw and ankle joint swelling, and determining lymphocyte infiltration. We further investigated the possible molecular mechanisms of curcumin’s therapeutic potential to inhibit the degradation of IκBα, the activation of NF-κB signaling pathway, the activity of NLRP3 inflammasome, and the mitochondrial damage in MSU crystal-stimulated inflammation. This study may provide new treatments for gout arthritis.

## Methods

### Mice

All mice were of C57BL/6 background and were housed with an alternating 12-h light/12-h dark cycle. All animal experiments were performed according to the Guidelines for the Care and Use of Laboratory Animals and were approved by the Ethics Committee of North Sichuan Medical College.

### Preparing MSU

One gram uric acid (Sigma-Aldrich, St. Louis, MO, USA) was dissolved in 200 ml of boiling water containing 6 ml of 1 N NaOH, adjusted to pH 8.9 through the addition of NaOH, and the mixture was crystallized at room temperature overnight. The precipitate was filtered from the solution and dried at 42 °C. The crystals were weighed under sterile conditions and suspended in PBS at a concentration of 25 mg/ml [16].

### Isolation of PBMCs, cell culture, and MSU stimulation

Fresh peripheral blood mononuclear cells (PBMCs) from healthy controls were plated at a density of 1 × 10^6^ cells/35-cm dishes with RPMI 1640 culture medium containing 10% fetal bovine serum. Curcumin was prepared according to the method described by Banerjee et al. [15]. Pretreatment with different concentrations of curcumin (1 μM, 5 μM, 10 μM) for 1 h in the dark before MSU suspension (0.2 mg/ml) was added to the culture plates, and the cells were cultured for 24 h at 37 °C in a 5% CO_2_ humidified incubator and collected for RNA extraction.

### THP-1 and RAW264.7 cell culture, MSU stimulation, and cytokine measurements

THP-1 cells were suspended in RPMI 1640 culture medium containing 10% fetal bovine serum and seeded in 24-well culture plates (2 × 10^5^ cells/ml/well). Phorbol myristate acetate (PMA; 100 ng/ml) was used to treat THP-1 cells for 48 h, and then THP-1-derived macrophages were obtained. RAW264.7 cells were cultured with DMEM containing 10% fetal bovine serum. THP-1 and RAW264.7 cells were pretreated with different concentrations of curcumin (1 μM, 5 μM, 10 μM) in the dark for 1 h and then stimulated with MSU suspension (0.2 mg/ml) for 24 h. In the THP-1 cells, the supernatants were used to detect cytokine levels and the levels of IL-1β, IL-6, TNF-α, and PGE2 were examined using ELISA (Neobioscience kit, Shenzhen, China) following the manufacturer’s guidelines.

### RNA extraction and real-time quantitative PCR analysis

RNA was extracted from THP-1 cells and PBMCs using the TRIzol method and was reverse-transcribed. The mRNA expression levels of IL-1β, IL-6, TNF-α, COX-2, TLR4, and NLRP3 were detected by SYBR green gene expression assays. The primer sequences are shown in Table 1.

**Table 1:** The primers used for quantitative PCR

	Forward sequence (5′–3′)	Reverse sequence (5′–3′)
IL-1β	ATGGCTTATTACAGTGGC	AACCAGCATCTTCCTCAG
IL-6	CCTTAGCCCTGGAACTGC	AAGGCAACTGGACCGAAG
TNF-α	CGAGTCTGGGCAGGTCTA	GGTTTCGAAGTGGTGGTC
COX-2	CTCAGACGCTCAGGAAAT	AGTTGAAGATTAGTCCGC
NLRP3	GCCTCAACAAACGCTACA	TGTTTTCCCAATCCCTGC
GAPDH	GTCACCAGGGCTGCTTTT	CTGGAAGATGGTGATGGG
MyD88	GGGTAGACCCACGAGTCC	TTCAAGAACAGAGACAGGCGG

### Total superoxide dismutase (T-SOD) activity assay

The supernatant of RAW264.7 cells lysates was collected for T-SOD detection using T-SOD assay kit (Jiancheng Technology Company, Nanjing, China). T-SOD activity was calculated relative to protein concentration.

### MitoTracker Green, MitoTracker Red, and Mito SOX Red label in the THP-1 and RAW264.7 cells

THP-1 and RAW264.7 cells were loaded with 100 nM green-fluorescing MitoTracker Green (MitoGreen, YEASEN Technology Company, Shanghai) for 30 min at 37 °C to measure mitochondrial content, and 500 nM MitoTracker Red (MitoRed, YEASEN Technology Company, Shanghai) for 30 min at 37 °C to detect mitochondrial membrane potential, followed by a wash with warmed complete culture medium. To examine mitochondrial reactive oxygen species (ROS) levels, cells were loaded with 5 μM MitoSOX Red (MitoSOX, YEASEN Technology Company, Shanghai) for 10 min at 37 °C, which is a mitochondrial superoxide indicator. The nucleus was stained with Hoechst 33342 for 10 min at 37 °C (Beyotime Technology Company, Beijing). The cells were live-imaged immediately after incubation with fresh complete culture medium for 60 min with the Olympus Laser Scanning Confocal Microscope. The excitation and emission wavelengths for each fluorescent dye were selected according to the manufacturer’s instructions. The fluorescence intensity of each tracer in various conditions was expressed as the fold of change versus control cells incubated in complete culture medium. All data were obtained from experiments with at least three replicates.

### Analyses of MSU-induced arthritis

According to previous reports, we selected moderate doses of curcumin to treat the mouse model of arthritis [14, 15]. Mice were pretreated with curcumin (150 mg/kg body weight, i.p. injection). After 1 h, 1 mg of MSU in 40 μl of PBS was injected into the ankle joint and footpad, and the same volume of PBS was simultaneously injected into the contralateral ankle joint and footpad as the control. The swelling index is expressed as the MSU-injected joint/PBS-injected joint ratio. Paw and ankle joint swelling was measured with an electronic caliper at the indicated time points. MSU suspensions were injected 24 h later, mice were sacrificed, footpad tissues were homogenized in RIPA buffer, and the supernatants were collected to detect the activity of myeloperoxidase (MPO) and the levels of related proteins. MPO activity, as a quantitative detection of neutrophil sequestration, was determined using an MPO colorimetric activity assay kit (Jiancheng Technology Co, Nanjing, China) in footpad tissue homogenates following the manufacturer’s instructions. Footpad tissues were fixed in 10% paraformaldehyde, and sections were stained with hematoxylin and eosin (HE) for histological analysis.

### Western blotting analysis

RIPA buffer was used to extract total protein from THP-1 cells or foot tissues. Cytosolic protein was extracted by Cytoplasmic Protein Extraction Kit (Beyotime, China). Nuclear protein extraction was performed using the CellLytic™ NuCLEAR™ Extraction Kit (Sigma, USA). The protein concentrations were measured using a BCA protein assay kit (Thermo Scientific, MA, USA). The protein samples were denatured by SDS-PAGE and transferred onto PVDF membranes. After blocking, the membranes were incubated with the primary antibodies at 4 °C overnight; anti-Phospho-NF-kB p105/50(Ser933) and anti-Phospho-NF-κB p65(Ser536) were obtained from Cell Signaling Technology (CST, USA); and anti-IκBα, anti-COX-2, anti-SOD2, anti-MyD88, anti-TLR4, anti-Caspase-1, anti-Lamin B1, anti-IL-1β, anti-NF-kB p105/50, anti- NF-κB p65, and anti-NLRP3 were obtained from HuaBio (Hangzhou, China). Then, the membranes were incubated with the secondary antibodies (1:5000) for 1 h at room temperature and exposed to the gel imaging system with a chemiluminescence kit. The band intensity was quantified using ImageJ software.

### Immunostaining

RAW264.7 cells were cultivated on cell slides, pretreated with curcumin (5 μM) for 1 h and then stimulated with MSU crystals for 24 h, fixed by 4% PFA. TritonX-100 (0.1%) was used to permeate for 10 min at room temperature. The cell slides were incubated with anti-Phospho-NF-kB p105/50(Ser933) and anti-Phospho-NF-κB p65 (Ser536) antibody (CST, USA) (1:100) at 4 °C overnight. After incubating with the secondary antibody, the images were detected by an Olympus Laser Scanning Confocal Microscope.

### Statistical analysis

GraphPad Prism 6 software was used for statistical analysis. All values were expressed as the mean ± SEM. A one-way ANOVA with Student’s *t* test with two or three repeats was used to determine the significant differences between groups. *P* < 0.05 was considered statistically significant.

## Results

### Curcumin inhibits the MSU-induced degradation of IκBα protein as well as NF-κB activation in THP-1-derived macrophages

**Fig. 1:**
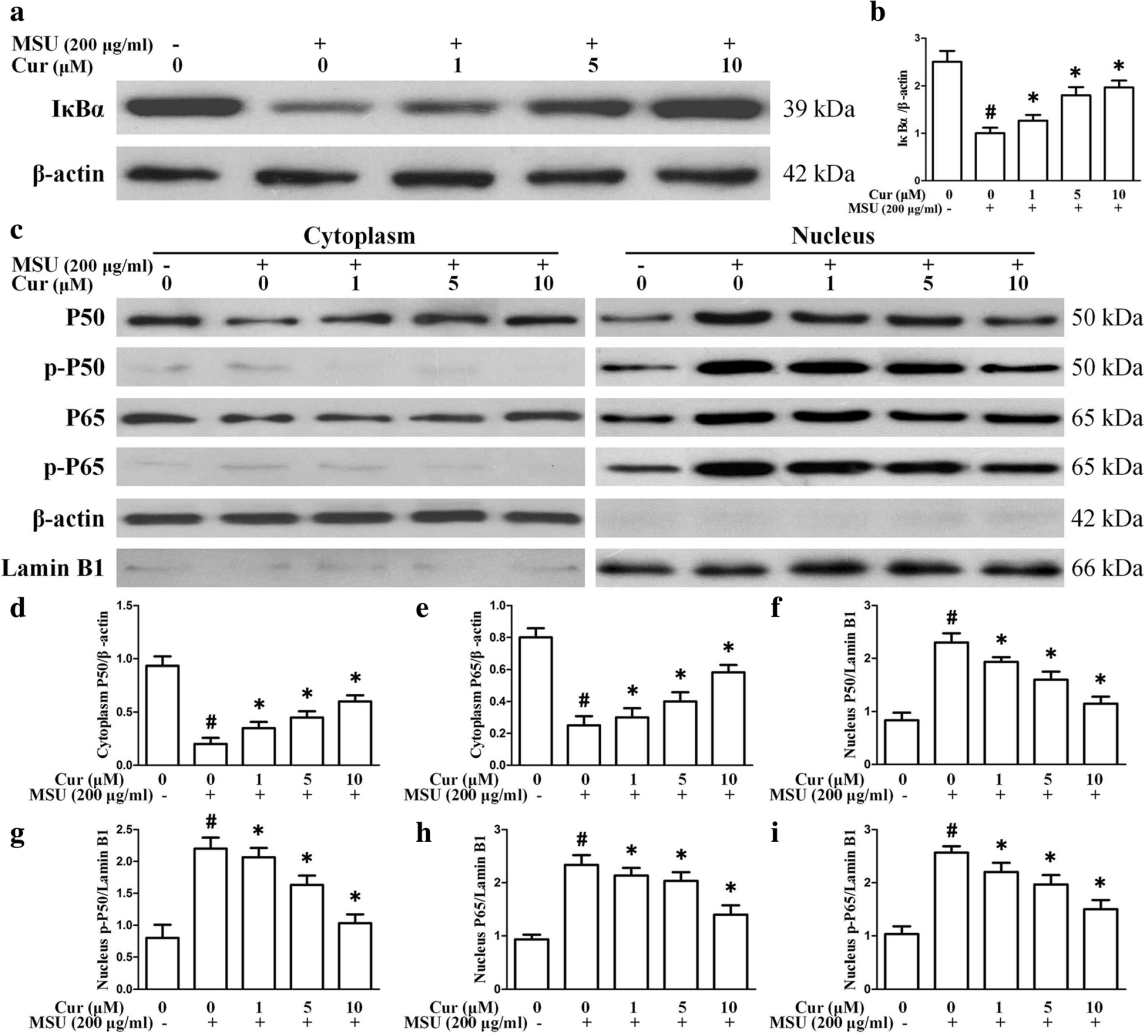
Effects of curcumin on MSU-induced degradation of IκBα and NF-κB activation in THP-1 cells. **a** THP-1 cells were pretreated with curcumin for 1 h and then stimulated with MSU suspensions. Total protein extracted from THP-1 cells was reacted with anti-IκBα by western blotting. **b** Densitometry analysis of IκBα. **c** THP-1 cells were pretreated with curcumin for 1 h and then stimulated with MSU suspensions. Cytosolic and nuclear protein extracted from THP-1 cells was respectively reacted with anti-phosphorylated P50 (p-P50), anti-phosphorylated P65 (p-P65), anti-P50, and anti-P65 by western blotting. **c–i** Densitometry analysis of p-P50, p-P65, P50, and P65 protein. The data represent the mean ± SEM for three experiments

**Fig. 2:**
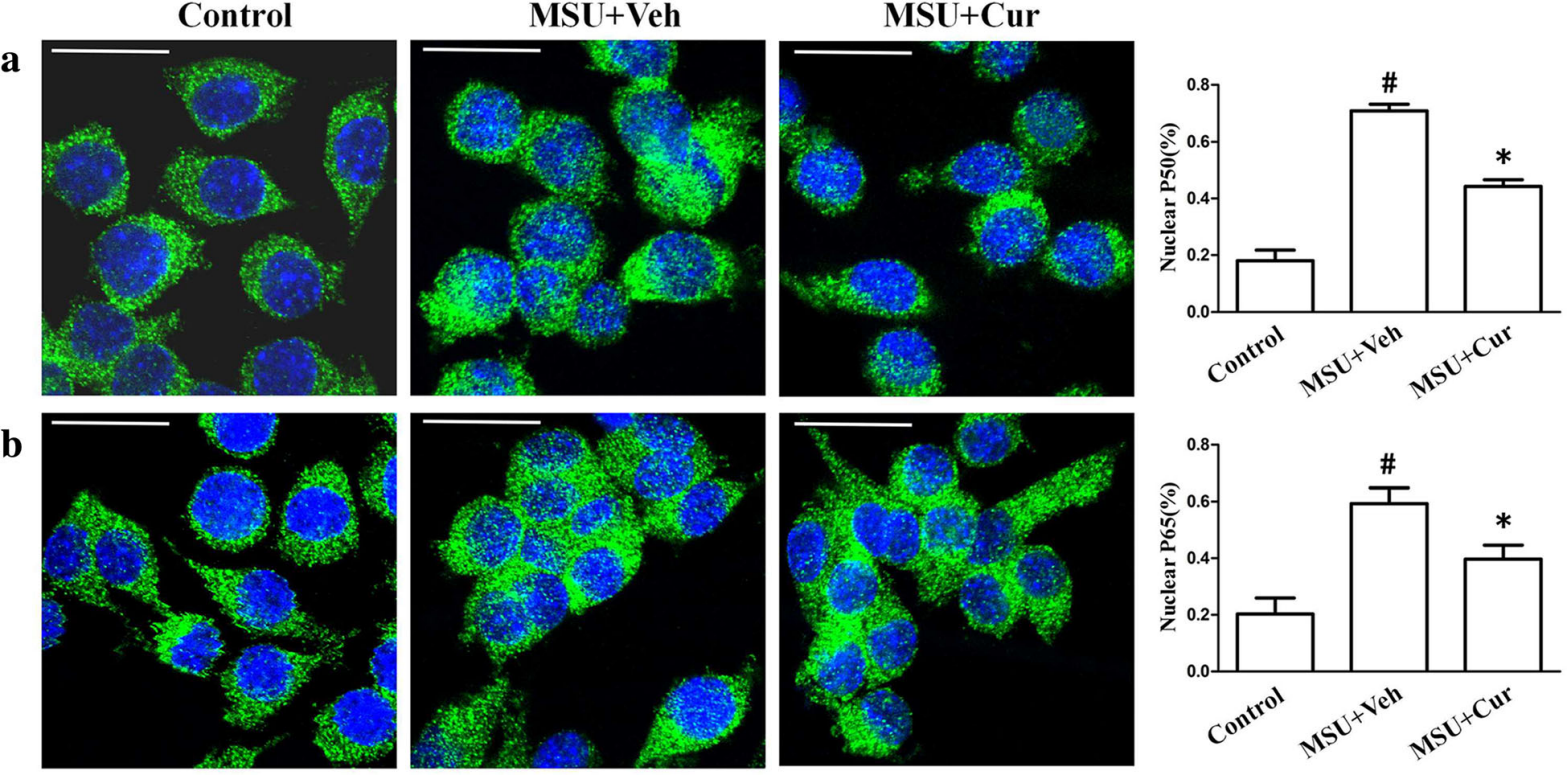
The effects of curcumin on the P50 and P65 nuclear translocation in MSU-stimulated RAW264.7 cells. **a** The percentages of p50 in the RAW264.7 nucleus were quantified in the absence (Control) or presence of MSU (0.2 mg/ml) or combinations (0.2 mg/ml MSU + 5 μM curcumin). **b** The percentages of p65 in the RAW264.7 nucleus were quantified in the absence (Control) or presence of MSU (0.2 mg/ml) or combinations (0.2 mg/ml MSU + 5 μM curcumin). Blue shows nuclei staining with DAPI. Scale bar: 10 μm. ^#^ Significantly different from absence both MSU and curcumin. * Significantly different from absence of curcumin, *P* < 0.05

NF-κB plays a key role in the pathogenesis of gout. To determine the effect of curcumin treatment on MSU-induced inflammation, we investigated the effect of curcumin on IκBα, an inhibitor of NF-κB. Our results indicated that accelerated degradation of IκBα and elevated expression of p-p65 and p-p50 was observed in MSU-stimulated THP-1 cells. As shown in Fig. 1 a and b, the basal level of IκBα was high in THP-1 cells, whereas it was substantially decreased in MSU-induced THP-1 cells and increased after curcumin treatment. Then, cytosolic and nuclear fractions were isolated from THP-1 cells and were respectively probed for the phosphorylation levels of NF-κB subunit p65 and P50 as well as p65 and p50 protein levels through western blotting. The results demonstrated that curcumin treatment was effective in downregulating the protein levels of p-p65 and p-p50 as well as p65 and p50 protein levels in the nucleus compared with the levels in the control (Fig. 1c–i). We further explored the effects of curcumin on the P50 and P65 nuclear translocation of RAW264.7 cells stimulated by MSU. The percentages of P50 and P65 nuclear translocation were significantly increased after MSU stimulation. Curcumin pretreatment reduced the relative contents of P50 and P65 in the THP-1 and RAW264.7 nuclei (Fig. 2).

### Curcumin blocks the expression of NF-κB downstream inflammatory cytokines, TLR4, MyD88, and NLRP3 in THP-1 cells and PBMCs

**Fig. 3:**
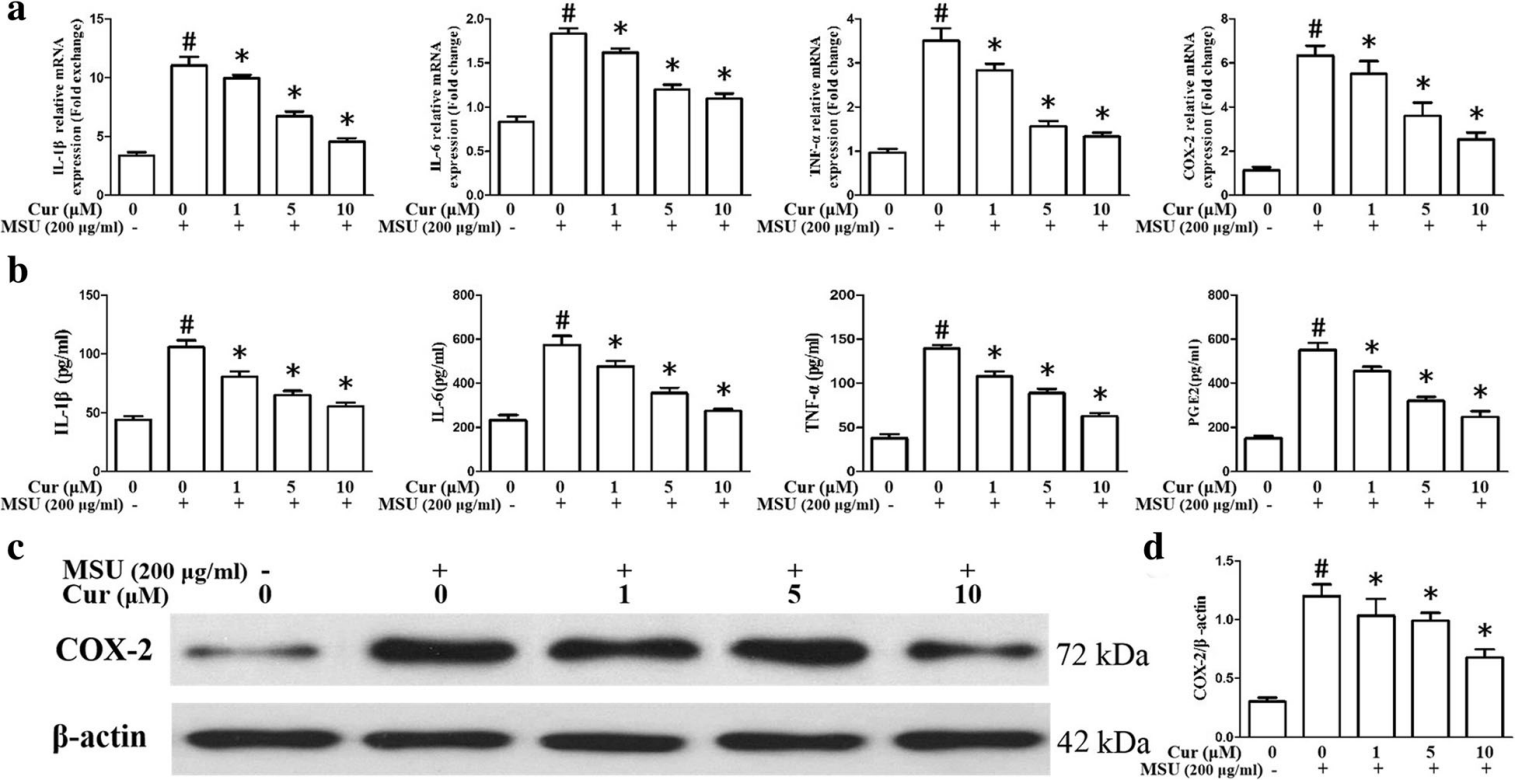
Effects of curcumin on the expression of MSU-induced NF-κB downstream inflammatory cytokines. **a** mRNA expression of the NF-κB downstream inflammatory cytokines IL-1β, IL-6, TNF-α, PGE2, and COX-2 in THP-1-derived macrophages treated with curcumin and MSU suspensions (*n* = 3, mean ± SEM). **b** Measurement of the secretion of the NF-κB downstream inflammatory cytokines IL-1β, IL-6, TNF-α, and PGE2 in macrophages treated with curcumin and MSU suspensions (*n* = 3, mean ± SEM). **c** Representative western blot analysis of COX-2 in MSU-stimulated THP-1-derived macrophages with pretreatment of curcumin. **d** Densitometry measurements of protein analysis. The data represent mean ± SEM for three experiments. ^#^Significantly different from absence both MSU and curcumin. *Significantly different from absence of curcumin, *P* < 0.05

**Fig. 4:**
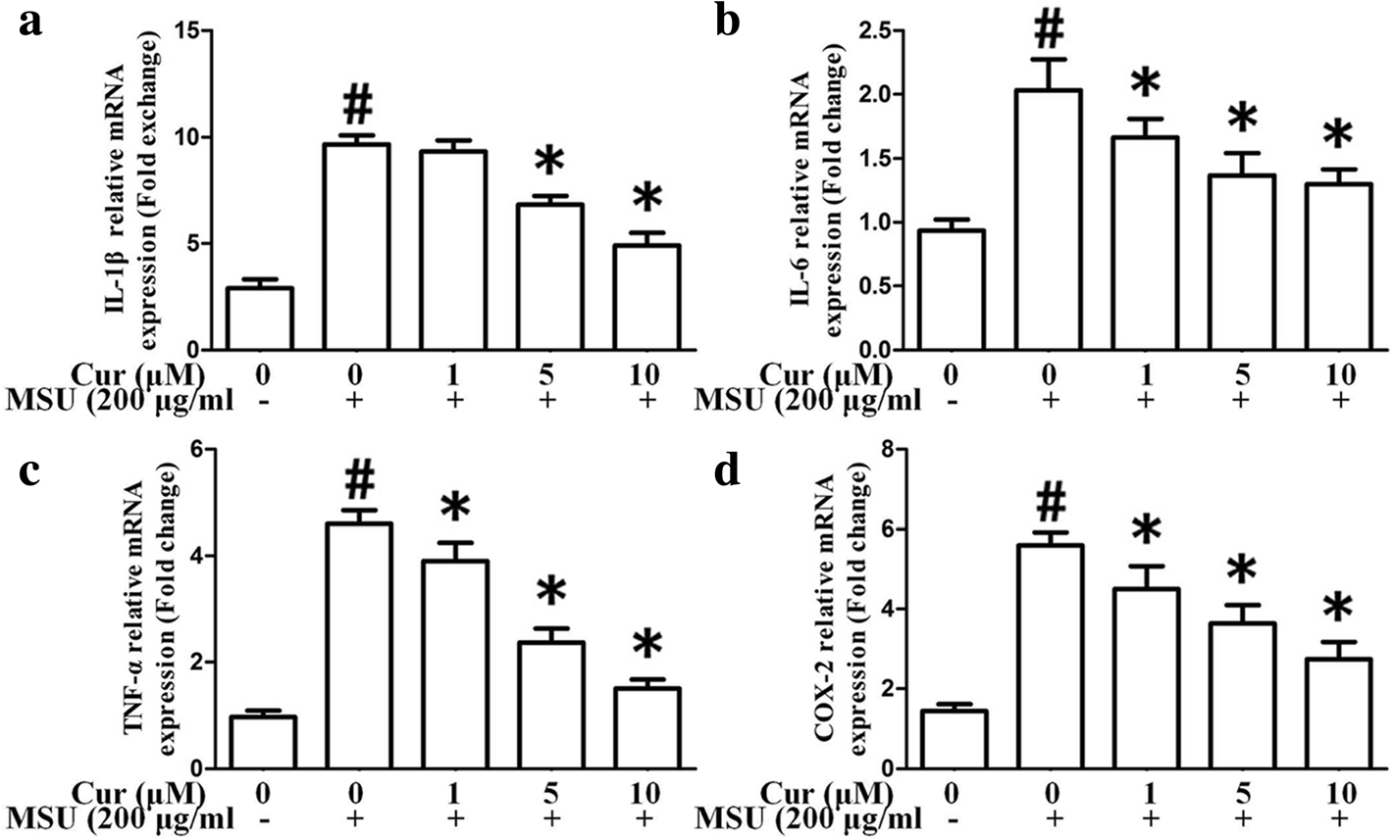
Curcumin inhibits the mRNA expression of MSU crystals induced inflammatory cytokines in the PBMCs. **a** The relative mRNA of IL-1β (*n* = 3, mean ± SEM). **b** The relative mRNA of IL-6 (*n* = 3, mean ± SEM). **c** The relative mRNA of TNF-α (*n* = 3, mean ± SEM). **d** The relative mRNA of COX-2 (*n* = 3, mean ± SEM). The data represent the mean ± SEM for three experiments. ^#^Significantly different from absence both MSU and curcumin. *Significantly different from absence of curcumin, *P* < 0.05

**Fig. 5:**
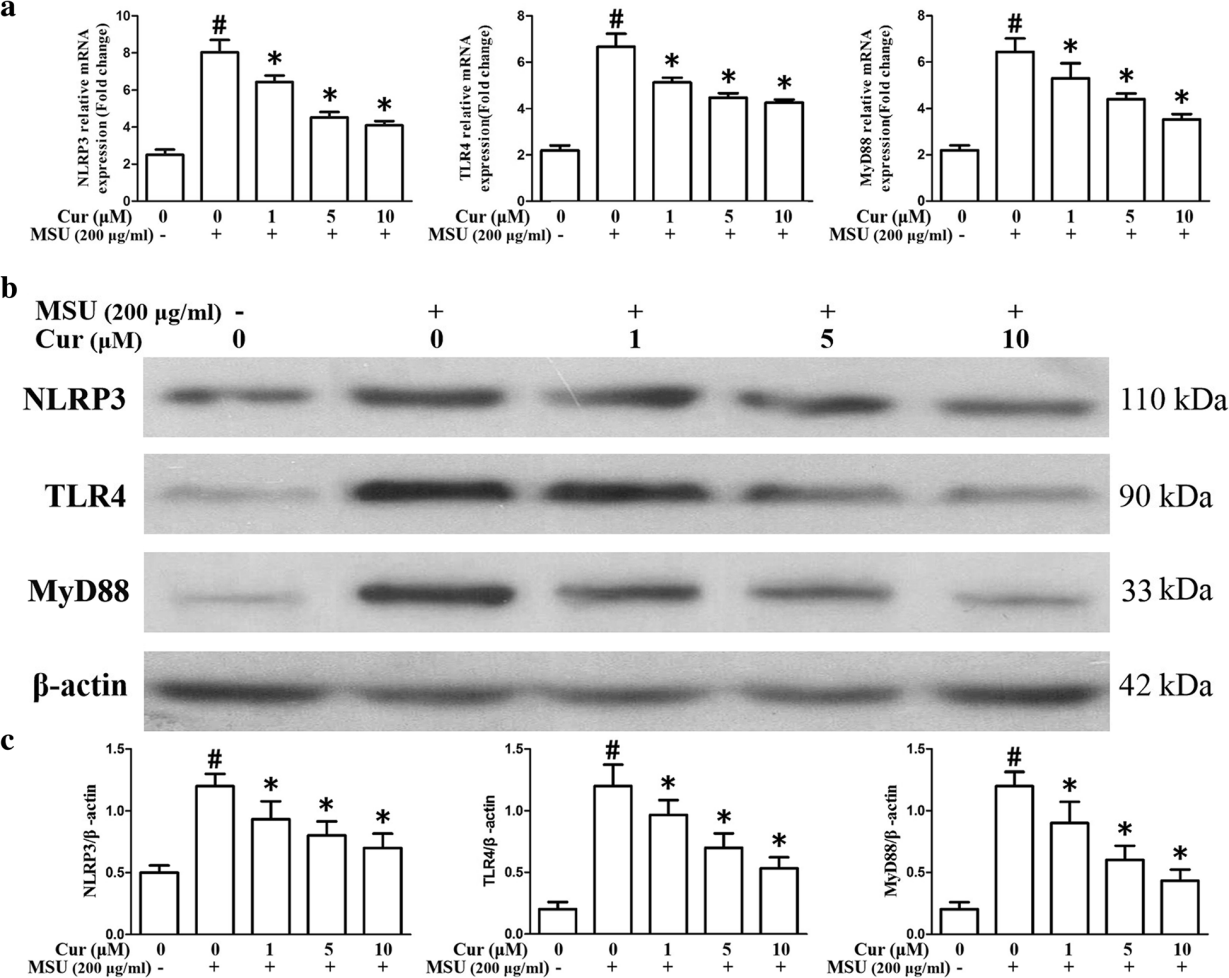
Curcumin blocks the expression levels of TLR4, MyD88, and NLRP3 in the MSU-induced THP-1 cells. **a** The relative mRNA expression of TLR4, MyD88, and NLRP3 in the MSU-stimulated THP-1 cells pretreated with curcumin (*n* = 3, mean ± SEM). **b** Effects of curcumin on the MSU-stimulated protein levels of TLR4, MyD88, and NLRP3 in the MSU-stimulated THP-1 cells; the protein levels were determined by western blot analysis. **c** Densitometry analysis of TLR4, MyD88, and NLRP3 protein. The data are expressed as mean ± SEM for three experiments. ^#^Significantly different from absence both MSU and curcumin. *Significantly different from absence of curcumin, *P* < 0.05

As shown in Fig. 3a, the mRNA expression levels of NF-κB-related inflammatory cytokine genes IL-1β, IL-6, TNF-α, and COX-2 were markedly enhanced as a result of MSU stimulation in the THP-1 cells. Nonetheless, the expression of these genes was significantly suppressed by curcumin treatment in a dose-dependent manner. In the PBMCs, pretreatment with curcumin also could inhibit the expression of inflammatory cytokine genes IL-1β, IL-6, TNF-α, and COX-2 (Fig. 4a–d). ELISA showed that the expression levels of IL-1β, IL-6, TNF-α, and prostaglandin E2 (PGE2) in culture supernatants from THP-1 cells were upregulated as a result of MSU stimulation, with a dose-dependent diminishment in MSU-induced inflammatory cytokines from THP-1 cells after curcumin administration (Fig. 3b). Consistent with the q-PCR and ELISA results, the western blotting analysis of the protein level of COX-2 improved after exposure to MSU in the THP-1 cells. Curcumin administration dramatically and dose-dependently reduced the expression level of COX-2 stimulated by MSU in the THP-1 cells (Fig. 3c, d). The effects on the expression of TLR4, MyD88, and NLRP3 in the THP-1 cells after curcumin administration were also evaluated by quantitative PCR and western blotting. The data indicated that in the presence of MSU, the expression levels of TLR4, MyD88, and NLRP3 were dramatically elevated; curcumin treatment reduced the expression levels of TLR4, MyD88, and NLRP3 in MSU-stimulated THP-1 cells (Fig. 5a–c).

### Curcumin inhibits NLRP3 inflammasome activation and relieves mitochondrial damage in murine macrophages and human monocytes

**Fig. 6:**
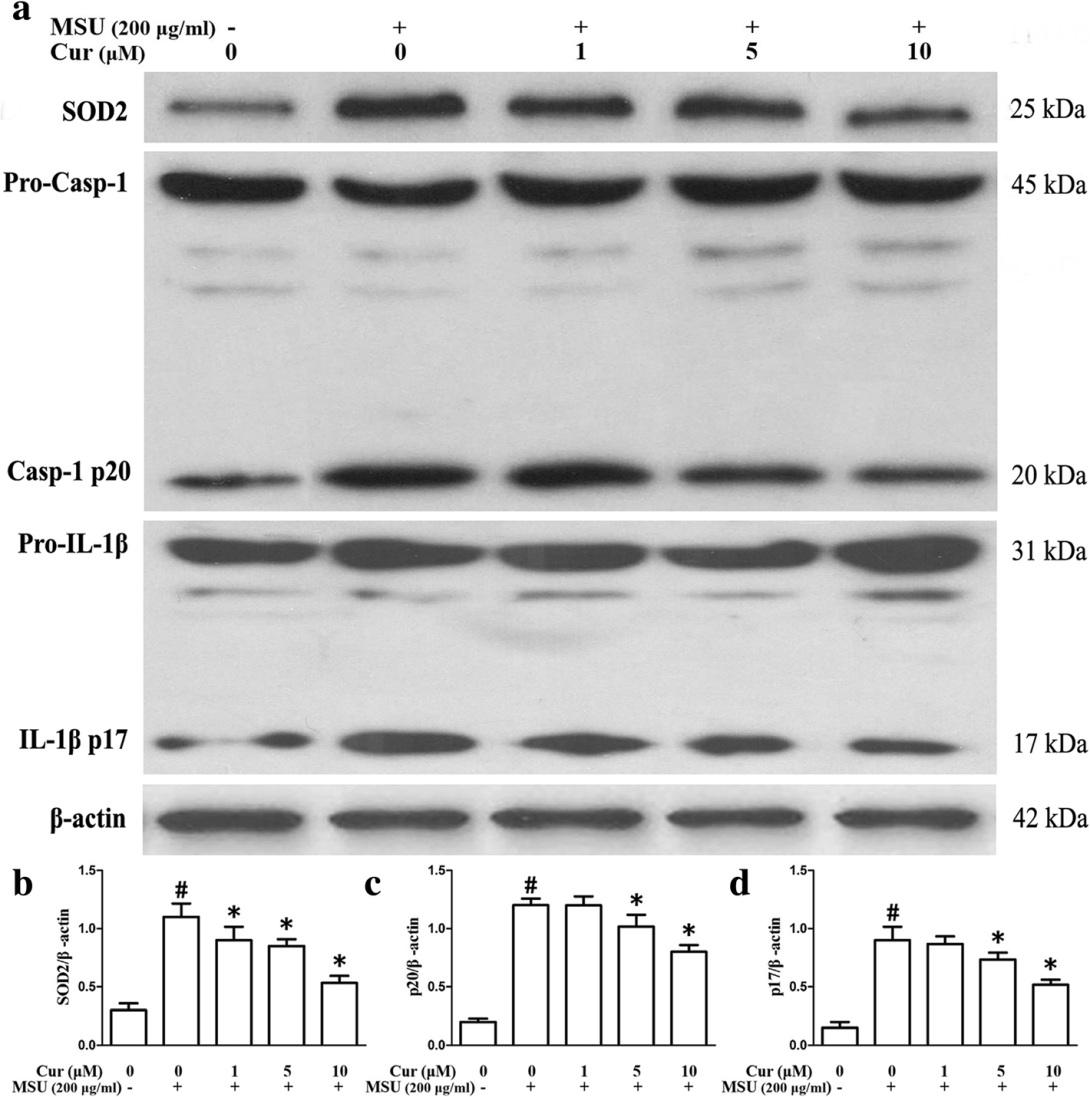
Curcumin suppresses the expression of SOD2, Caspase-1 activation, and IL-1β maturation in the THP-1 cells. **a** Effects of curcumin on the MSU-stimulated expression levels of SOD2, p20 subunit of Caspase-1, and active p17 form of IL-1β; the expression levels were analyzed by western blot. **b–d** Densitometric analysis was used to quantify the level of SOD2, cleavage of caspase-1, and active p17 form of IL-1β. The data are expressed as mean ± SEM for three experiments. ^#^Significantly different from absence both MSU and curcumin. *Significantly different from absence of curcumin, *P* < 0.05

**Fig. 7:**
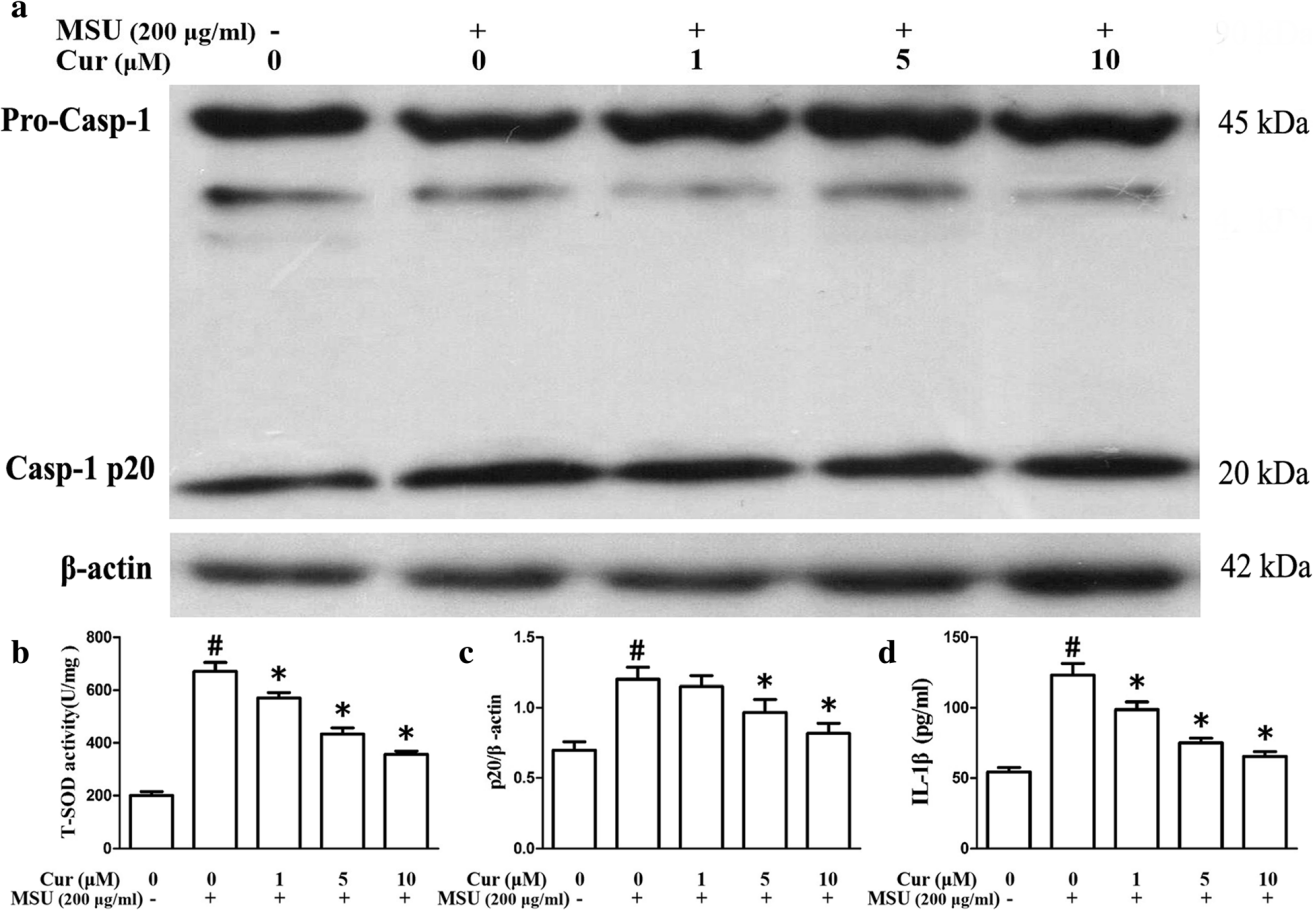
Curcumin inhibits the activity of T-SOD, Caspase-1 activation, and secretion of IL-1β in the MSU-stimulated RAW264.7 cells. **a** Effects of curcumin on the MSU-stimulated protein levels of p20 subunit of Caspase-1. **b** the activity of T-SOD (*n* = 3, mean ± SEM). **c** Densitometry analysis of p20 subunit of Caspase-1, the results represent the mean ± SEM for three experiments. *P* < 0.05. **d** Supernatants were analyzed by ELISA for IL-1β (*n* = 3, mean ± SEM). ^#^Significantly different from absence both MSU and curcumin. *Significantly different from absence of curcumin, *P* < 0.05

To test whether curcumin has an influence on the NLRP3 inflammasome activation in the MSU-induced inflammation, we detected whether curcumin could suppress Caspase-1 cleavage and IL-1β secretion. THP-1 and RAW264.7 cells were pretreated with curcumin for 1 h and then stimulated them with MSU crystals. Both Caspase-1 activation and IL-1β maturation were measured using immunoblots that detect the enzymatically active p20 subunit of Caspase-1 and the biologically active p17 form of IL-1β in the THP-1 cells, respectively. In the RAW264.7 cells, caspase-1 activation was examined through immunoblots, and the secretion of IL-1β was measured using ELISA. These data indicated that curcumin dose-dependently impeded MSU-induced cleavage of caspase-1 into p20 and IL-1β maturation in both THP-1 cells (Fig. 6a, c, d) and RAW264.7 cells (Fig. 7a, c, d).

**Fig. 8:**
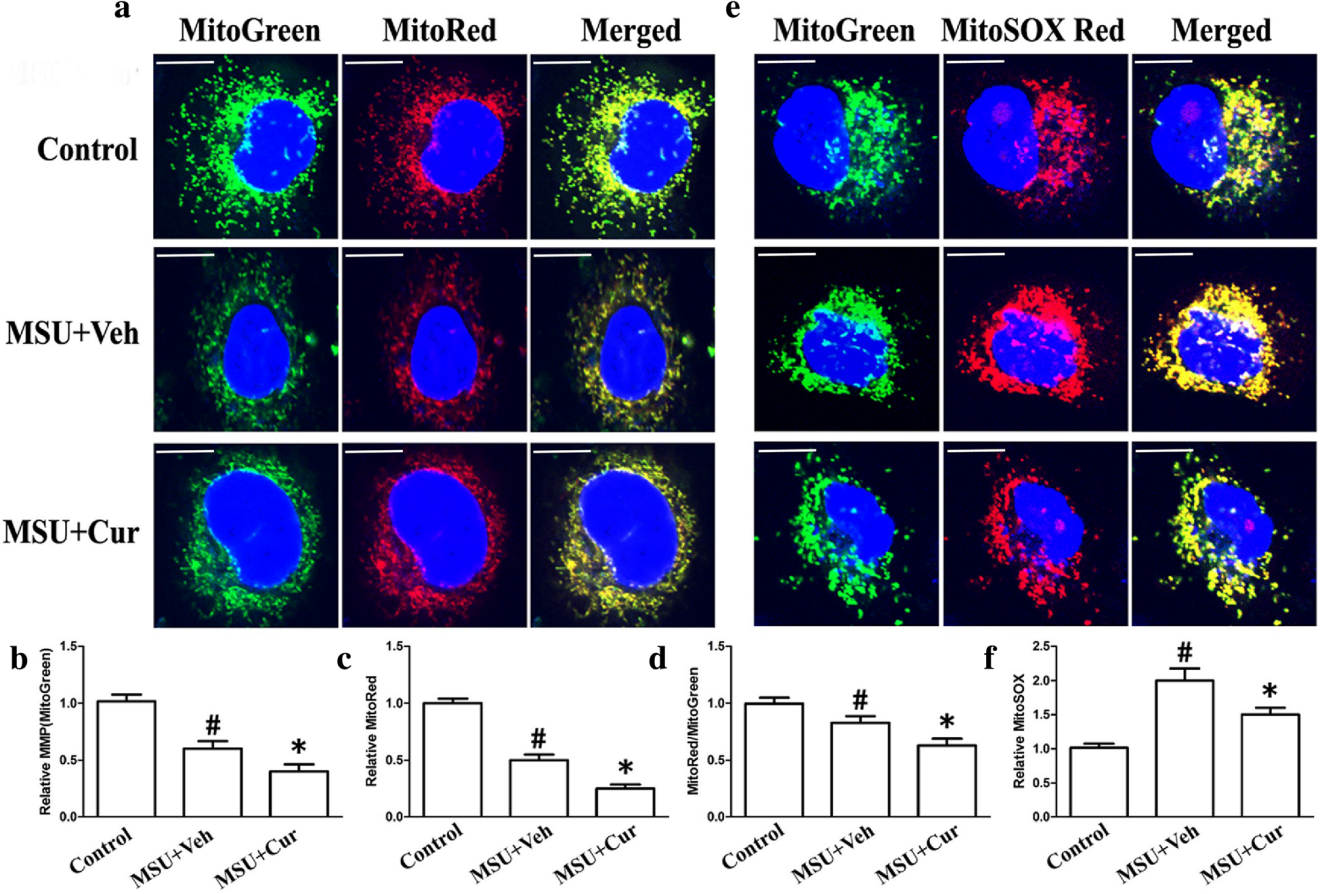
Curcumin blocks the decrease of mitochondrial membrane potential and reduces the production of mitochondrial ROS in the MSU-stimulated THP-1 cells. **a** Representative micrographs of MitoTracker Red- and MitoTracker Green-labeled THP-1 cells in the absence (Control) or presence of MSU (0.2 mg/ml) or combinations (0.2 mg/ml MSU + 5 μM curcumin); blue shows nuclei staining with Hoechst 33342. Scale bar: 10 μm. **b** The relative fluorescence intensities of MitoTracker Green were quantified in MSU-treated THP-1 cells which were divided by the fluorescence intensities of MitoTracker Green in the MSU-untreated THP-1 cells. **c** The relative fluorescence intensities of MitoTracker Red were quantified in MSU-treated THP-1 cells which were divided by the fluorescence intensities of MitoTracker Green in the MSU-untreated THP-1 cells. **d** The ratio of relative fluorescence intensities of MitoTracker Red to the relative fluorescence intensities of MitoTracker Green. **e** Representative micrographs of MitoSOX Red- and MitoTracker Green-labeled THP-1 cells in the absence (Control) or presence of MSU (0.2 mg/ml) or combinations (0.2 mg/ml MSU + 5 μM curcumin). **f** The relative fluorescence intensities of MitoSOX Red were quantified in MSU-treated THP-1 cells which were divided by the fluorescence intensities of MitoSOX Red in the MSU-untreated THP-1 cells; blue shows nuclei staining with Hoechst 33342. For all measurements, the values were from three independent experiments with four fields of view in each experiment

**Fig. 9:**
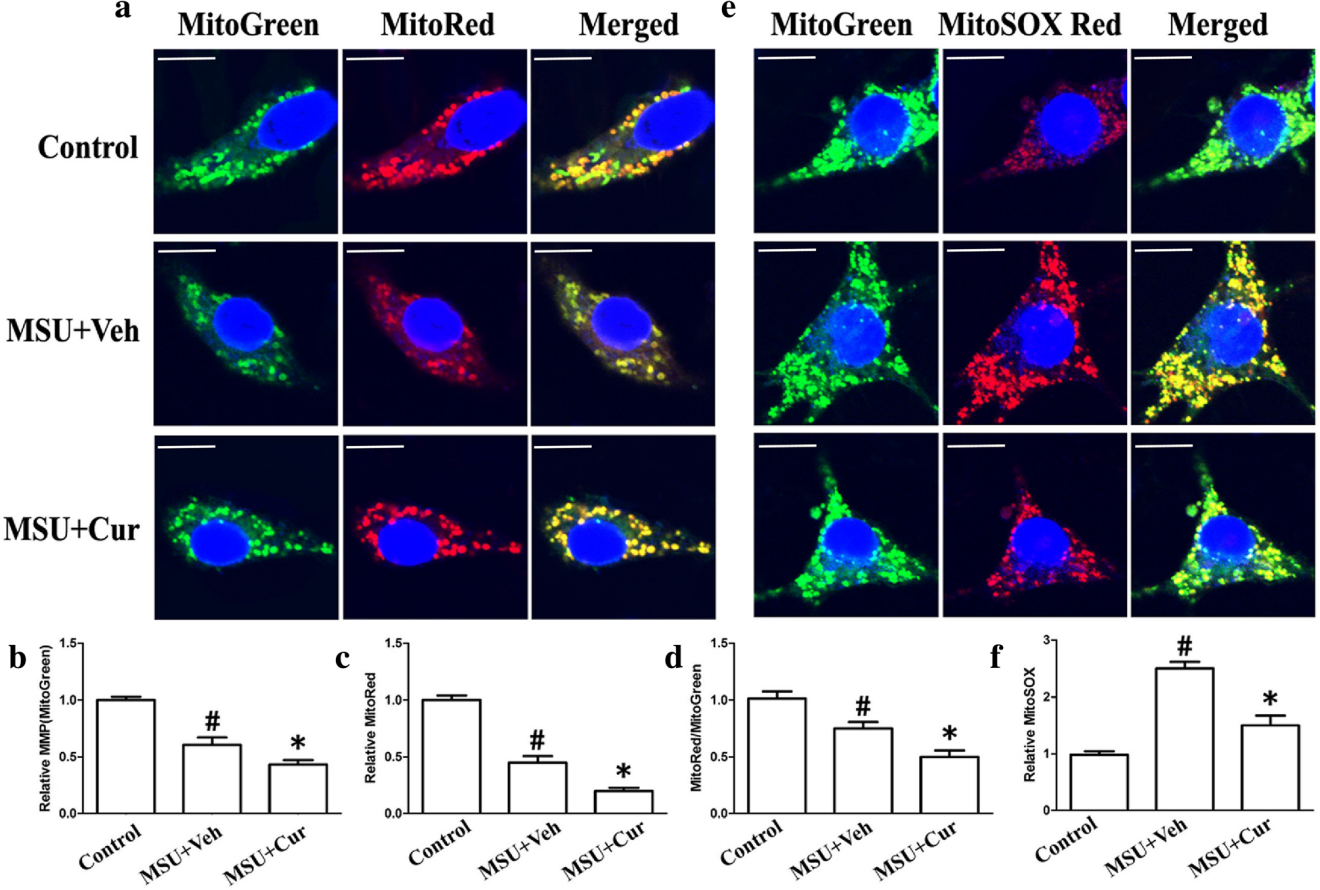
Curcumin prevents the mitochondrial MMP collapse and lowers the production of mitochondrial ROS in the MSU-stimulated RAW264.7 cells. **a** Representative micrographs of MitoTracker Red- and MitoTracker Green-labeled THP-1 cells in the absence (Control) or presence of MSU (0.2 mg/ml) or combinations (0.2 mg/ml MSU + 5 μM curcumin); blue shows nuclei staining with Hoechst 33342. Scale bar: 10 μm. **b** The relative fluorescence intensities of MitoTracker Green were quantified in MSU-treated THP-1 cells which were divided by the fluorescence intensities of MitoTracker Green in the MSU-untreated THP-1 cells. **c** The relative fluorescence intensities of MitoTracker Red were quantified in MSU-treated THP-1 cells which were divided by the fluorescence intensities of MitoTracker Green in the MSU-untreated THP-1 cells. **d** The ratio of relative fluorescence intensities of MitoTracker Red to the relative fluorescence intensities of MitoTracker Green. **e** Representative micrographs of MitoSOX Red- and MitoTracker Green-labeled THP-1 cells in the absence (Control) or presence of MSU (0.2 mg/ml) or combinations (0.2 mg/ml MSU + 5 μM curcumin). **f** The relative fluorescence intensities of MitoSOX Red were quantified in MSU-treated THP-1 cells which were divided by the fluorescence intensities of MitoSOX Red in the MSU-untreated THP-1 cells; blue shows nuclei staining with Hoechst 33342. All the values were from three independent experiments with four fields of view in each experiment

Many studies have been proposed that mitochondrial damage is an important signal responsible for NLRP3 inflammasome activation. The main characteristics of mitochondrial damage are the production of ROS, lower mitochondrial membrane potential (MMP), and a reduction of mitochondrial content. We assessed mitochondrial function in both THP-1 and RAW264.7 cells that were incubated with MSU crystals in the presence or absence of curcumin by measuring MMP through using MitoTracker Red (MitoRed). Total mitochondrial content was determined using MitoTracker Green (MitoGreen). After MSU stimulation, both THP-1 cells and RAW264.7 cells showed a lower MMP and decreased total mitochondrial content (Fig. 8a–c, THP-1 cells; Fig. 9a–c, RAW264.7 cells). Curcumin administration elevated both MMP and total mitochondrial content in the THP-1 cells and RAW264.7 cells (Fig. 8a–c, THP-1 cells; Fig. 9a–c, RAW264.7 cells). The ratio of MitoRed to MitoGreen, which reflects levels of polarized functional mitochondria [24], was significantly lower in MSU-stimulated cells than in controls, supporting that MSU stimulation impaired mitochondrial function (Fig. 8d, THP-1 cells; Fig. 9d, RAW264.7 cells). Pretreatment with curcumin increased the ratio of MitoRed to MitoGreen in the THP-1 and RAW264.7 cells, which suggested that curcumin could preserve the mitochondrial function upon MSU crystal stimuli (Fig. 8d, THP-1 cells; Fig. 9d, RAW264.7 cells).

Mitochondrial dysfunction can lead to oxidative stress and production of reactive oxygen species (ROS). Therefore, MitoSOX Red was used to detect the mitochondrial ROS levels of THP-1 and RAW264.7 cells. MitoSOX Red is a mitochondrial superoxide indicator inserted into mitochondrial DNA during oxidation, generating Red fluorescence. Consistent with recent data, THP-1 and RAW264.7 cells pretreated with curcumin displayed decreased MitoSOX Red fluorescence upon MSU crystal stimuli, indicating lower levels of superoxide production (Fig. 8e, f, THP-1 cells; Fig. 9e, f, RAW264.7 cells). We further noted that the total activity of T-SOD (Fig. 7b) and protein levels of mitochondrial matrix protein SOD2 (superoxide dismutase 2, mitochondrial) (Fig. 6a) also decreased in the RAW264.7 cells, which further indicated that curcumin could inhibit the production of mitochondrial ROS.

### Curcumin reduces the severity of MSU-induced arthritis

**Fig. 10:**
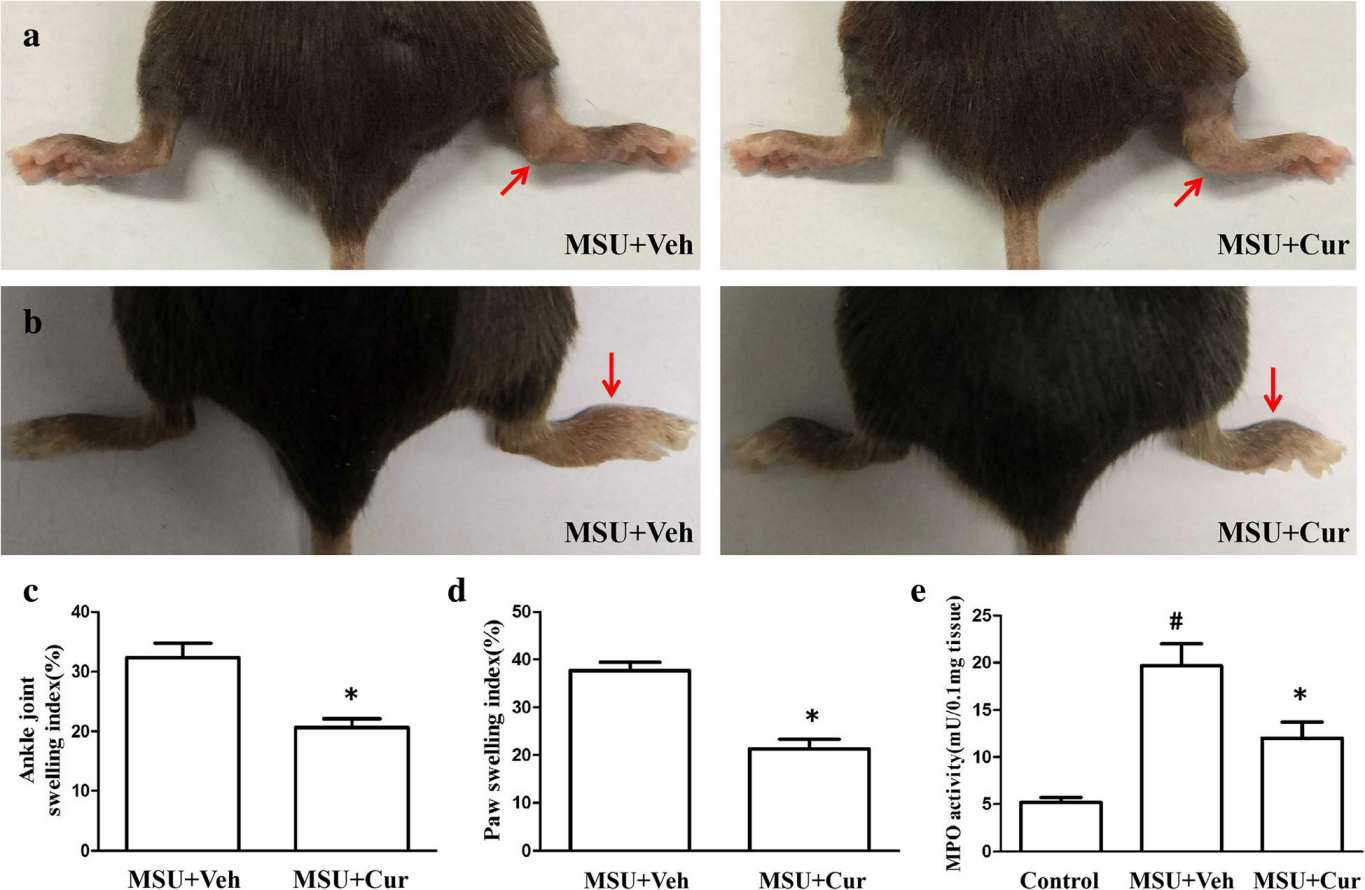
Curcumin alleviates the swelling of ankle and footpad in MSU-induced arthritis. **a** MSU suspensions were injected into the right rear ankle joints of C57BL/6 mice, while the same volume of PBS was injected into the left ankles. The swelling is expressed as the right-left/left ratio and a ratio > 0.15 indicates inflammation. Data are expressed as the mean ± SEM of six mice per group. **b** A total of 1 mg/40 μl MSU suspensions were injected into the right footpad of mice, and foot thickness was detected 24 h after MSU administration (*n* = 6 for each group). **c** The ankle joint swelling index is expressed as the MSU-injected joint/PBS-injected joint ratio. **d** The paw swelling index is expressed as the MSU-injected paw/PBS-injected paw ratio. **e** Myeloperoxidase (MPO) activity of the homogenates of footpad tissue (*n* = 4 per group, mean ± SEM)

**Fig. 11:**
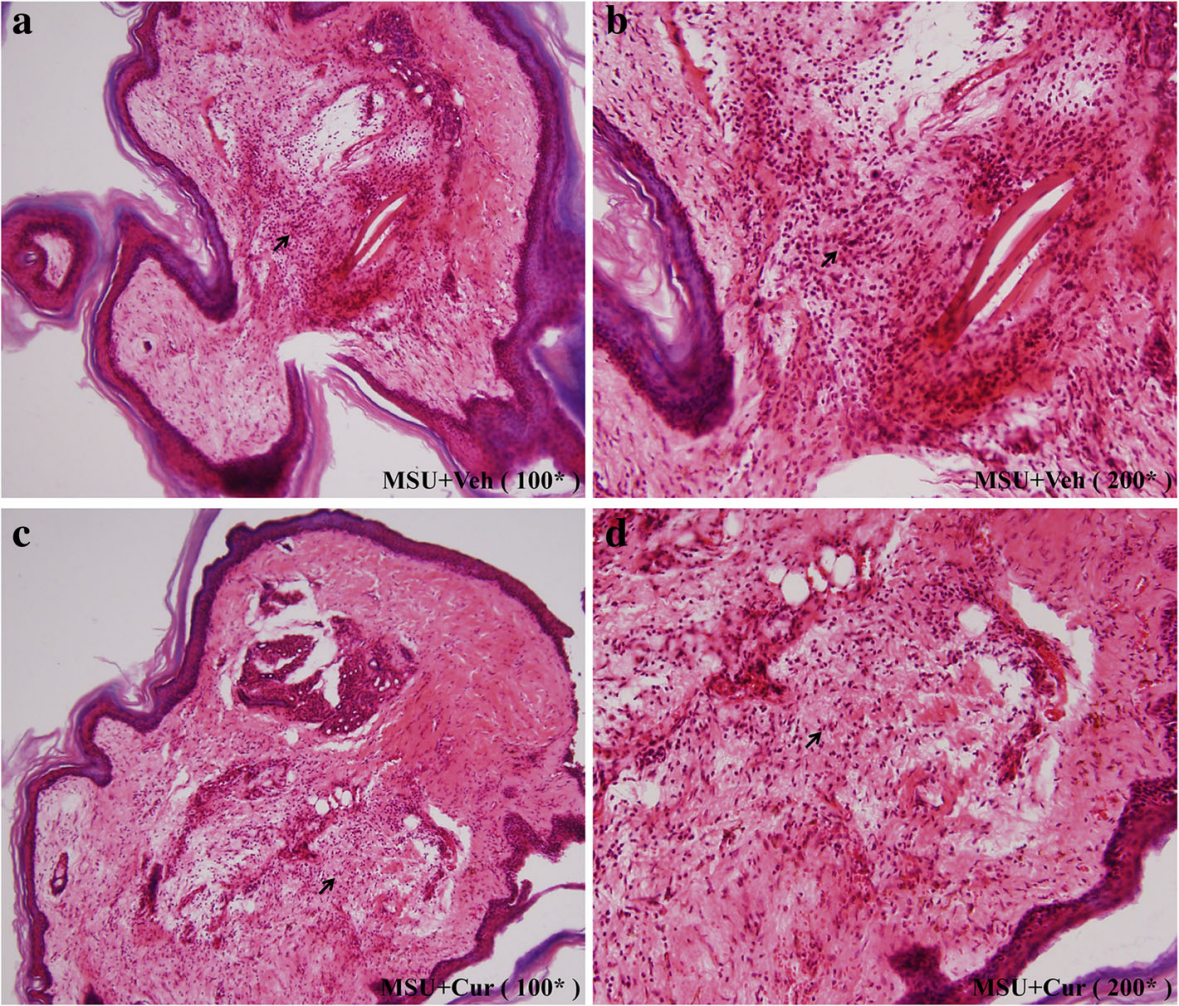
Curcumin inhibits the inflammatory cells infiltration in the MSU injection of mice footpad. **a** Representative photographs of HE staining of footpads from the MSU + vehicle group (× 100 original magnification). **b** MSU + vehicle group (200 × original magnification), arrow indicates abundant inflammatory cells in the footpad tissue section. **c** Representative photographs of HE staining of footpads from the MSU + curcumin group (× 100 original magnification). **d** MSU + curcumin group (× 200 original magnification), arrow indicates fewer inflammatory cells in the footpad tissue section

MSU suspensions were injected into the footpad and ankle joint of mice to simulate the etiologic origin of human gouty arthritis. We investigated the role of curcumin in the model of gouty arthritis induced by MSU. Greatly increased ankle swelling was observed after injection of MSU crystals into the footpad (Fig. 10a, c). IP treatment with curcumin (150 mg/kg) resulted in a significant reduction in ankle swelling compared with the ankle swelling in the vehicle-treated group. Consistent with the observed reduction in ankle swelling, curcumin treatment also significantly suppressed the paw swelling caused by the MSU crystals (Fig. 10b, d). Histological analyses of the footpad tissues showed that there were many lymphocyte infiltrations in the section of footpad tissues (Fig. 11a, b). Curcumin treatment visibly suppressed the influx of inflammatory cells, which were mostly neutrophils (Fig. 11c, d). Through the analysis of the activity of myeloperoxidase (MPO) in the homogenate of the footpad tissues, the recruitment of neutrophils induced by MSU crystals was analyzed, and the results are shown in Fig. 10e. Curcumin effectively reduced the MPO activity induced by MSU crystals (Fig. 10e). These results suggest that curcumin administration alleviates the acute gout symptoms caused by MSU crystals.

### Effects of curcumin on the degradation of IκBα, the activation of NF-κB, and the expression of NLRP3 in the gouty arthritis mice model

**Fig. 12:**
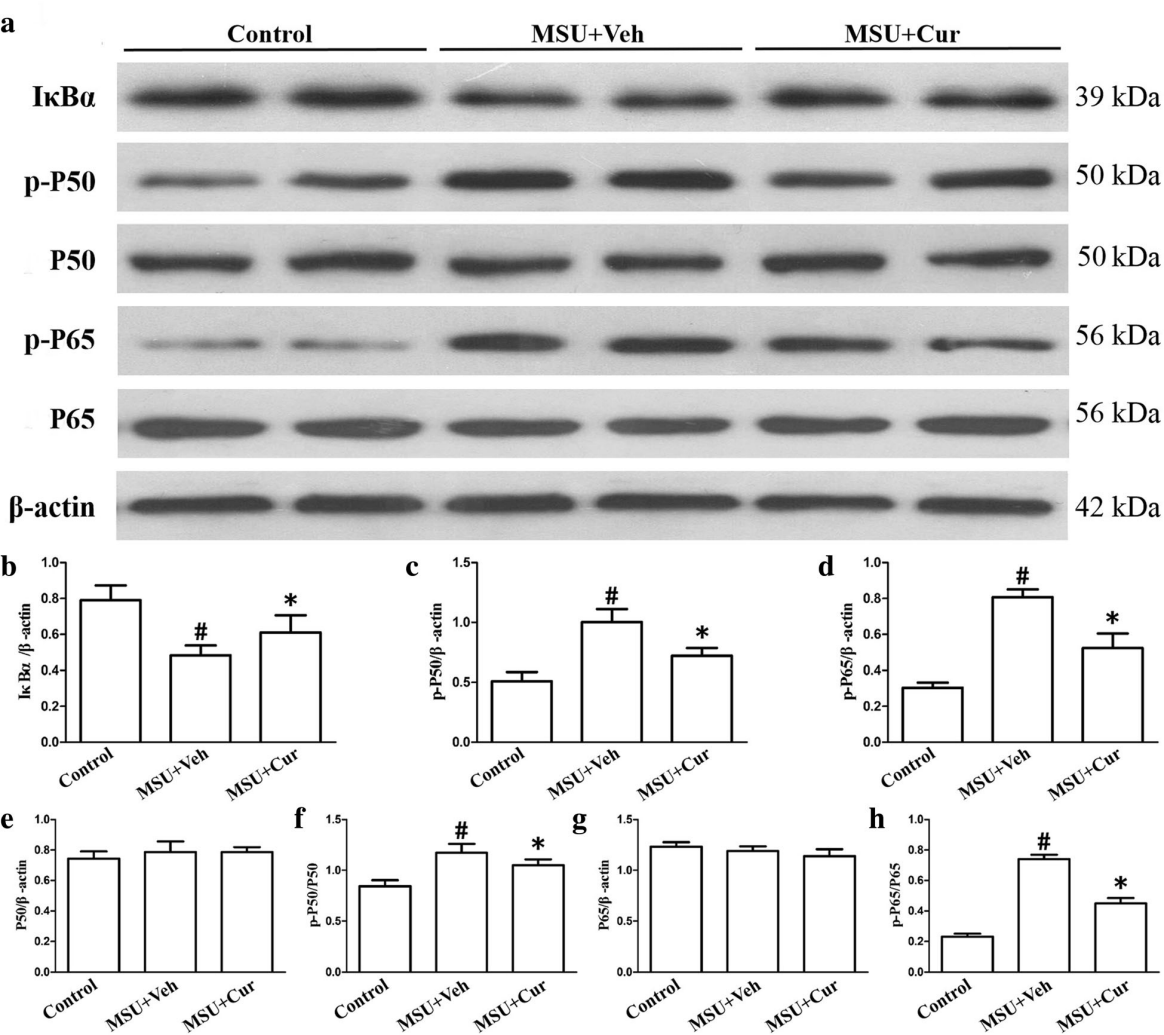
Curcumin blocks the MSU-induced degradation of IκBα and NF-κB activation in the footpad tissue of mice with MSU-induced arthritis. **a** The total protein extracted from the footpad tissue was analyzed by immunoblotting for degradation of IκBα and NF-κB activation. **b–h** Densitometry measurements of protein analysis. The data represent the mean ± SEM of four mice per group. ^#^Significantly different from vehicle (Veh) alone, *P* < 0.05. *Significantly different from MSU alone, *P* < 0.05

**Fig. 13:**
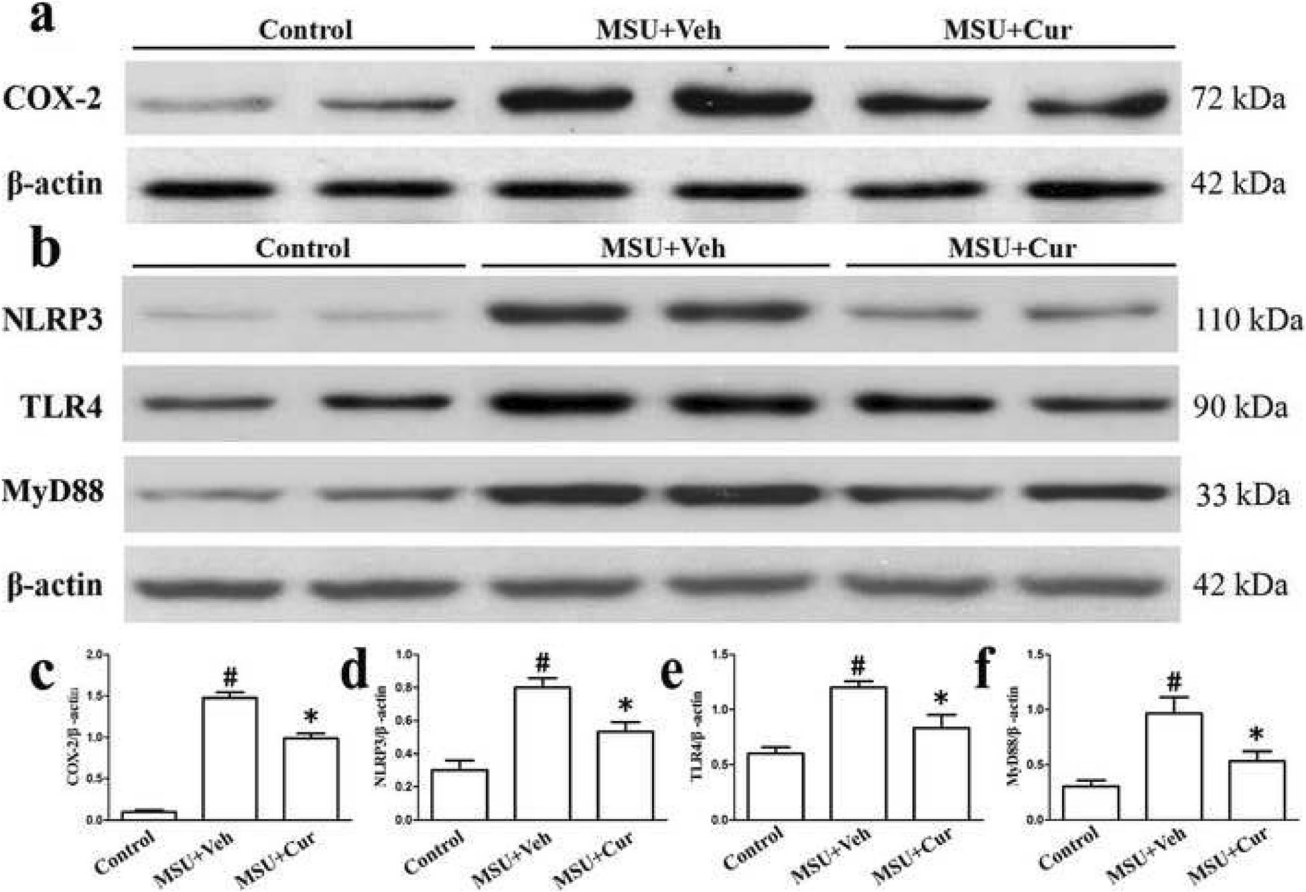
Curcumin suppresses the protein levels of MSU-induced COX-2, TLR4, MyD88, and NLRP3 in the footpad tissue of mice with MSU-induced arthritis. **a** Total protein was examined by immunoblotting for the protein level of COX-2. **b** Total protein was examined by immunoblotting for the protein levels of TLR4, MyD88, and NLRP3. **c**, **d**, **e**, **f** Densitometry analysis of TLR4, MyD88, and NLRP3. The data are expressed as the mean ± SEM of four mice per group. ^#^Significantly different from vehicle (Veh) alone, *P* < 0.05. *Significantly different from MSU alone, *P* < 0.05

Twenty-four hours after the MSU suspensions were injected into the footpads with or without curcumin, the degradation of IκBα, the activation of NF-κB, and the expression of COX-2, TLR4, MyD88, and NLRP3 were determined by western blotting in the footpad tissue extracts. The results (Fig. 12a–h) indicated that MSU could stimulate the degradation of an inhibitor of NF-kB (IκBα), while promoting the activation of NF-κB. COX-2, as a target gene downstream of NF-κB, MSU also accelerated COX-2 expression. However, after curcumin treatment, the protein level of IκBα was markedly increased; the total protein levels of P65 and P50 did not change, and there was a decrease in the phosphorylation levels of p-p65 and p-p50. Curcumin also suppressed the expression of COX-2 (Fig. 13a, c). NLRP3 is a component of inflammasome, and curcumin could inhibit the protein level of NLRP3 (Fig. 13b, d). TLR4, at the plasma membrane, interacts with MSU crystals. TLR4 expression improved significantly after MSU administration, and curcumin treatment attenuated MSU-stimulated TLR4 expression (Fig. 13b, e). TLR4 signaling involves the recruitment of adapter protein MyD88 and ultimate activation of NF-kB, and curcumin also lowered the expression of MyD88 because of MSU crystals stimuli (Fig. 13b, f).

## Discussion

The incidence of gout is on the rise globally, both in developed and developing countries. Acute gout is characterized by attacks of severe pain, stiffness, and swelling of a distal joint, which seriously affects the quality of life of patients [25]. The objective of this study was to investigate the therapeutic effects of curcumin on MSU crystal-induced inflammation through the suppression of the degradation of IκBα, the activity of NF-κB signaling pathway, the NLRP3 inflammasome activation, and the mitochondrial damage.

The activation of NF-κB signaling is the core mediator of the inflammatory response [26]. Following the degradation of inhibitory protein IκBα in the proteasome, the NF-κB signaling pathway was activated and then exerted a proinflammatory role by regulating the expression of downstream genes, such as proinflammatory cytokines [27, 28]. Disordered NF-κB activation is closely associated with MSU crystal-induced inflammation. NF-κB acts as a molecular target for curcumin activity. The detection of the expression of NF-κB signaling pathway-related genes is very important for the study of curcumin in the treatment of gout arthritis.

In our study, we first explored the anti-inflammatory effects of curcumin on MSU crystal-induced inflammation in macrophages. Three critical proteins in the NF-κB signaling axis were detected by using western blotting in THP-1 cells. The degradation of IκBα was notably increased in the presence of MSU, and curcumin inhibited IκBα degradation in an almost dose-dependent manner. The protein levels of p-p65 and p-p50 were significantly reduced after curcumin treatment, indicating that curcumin attenuated NF-κB activation. The expression levels of IL-1β, IL-6, TNF-α, COX-2, and PGE2 are regulated by NF-κB signaling [29–31]. Curcumin treatment inhibited the expression of IL-1β, IL-6, TNF-α, COX-2, and PGE2 in a dose-dependent manner through the inhibition of NF-κB activation, which alleviated MSU-induced inflammation in THP-1 cells. A previous study demonstrated that TLR4 mediated the initial steps of gout pathogenesis and that TLR4 expression was pivotal to MSU crystal-induced inflammation [32]. Our data indicated that curcumin repressed MSU-stimulated expression of TLR4. In the TLR4 signaling pathway, MyD88-dependent signaling pathway is a vital activator of NF-κB, and curcumin also could inhibit the expression of MSU-induced MyD88 in the THP-1 cells. The NLRP3 inflammasome is an important innate immune pathway that regulates the release of inflammatory cytokines, which are activated by various bacterial toxins and crystals [33, 34]. The NLRP3 inflammasome consists of NLRP3, apoptosis-associated speck-like protein containing a caspase activation recruitment domain (ASC) and precursor caspase-1 (pro-caspase1). In the present study, curcumin affects not only the expression of NLRP3, but also the activity of NLRP3 inflammasome.

Mitochondrial damage plays a critical role in the activation of the NLRP3 inflammasome. Our data indicated that curcumin could prevent mitochondrial damage, which was not consistent with a previous report [14], because in our study the final concentration of curcumin was kept between 1 and 10 μM to avoid aggregation, and curcumin solution was prepared fresh every time in the dark. In our present findings, the effect of curcumin on mitochondrial function was evaluated by examining the membrane potential of mitochondria. Curcumin treatment could block the decrease of mitochondrial membrane potential, which suggested that curcumin has a protective effect on mitochondrial function. A previous study has suggested a crosstalk between mitochondrial dysfunction and oxidative stress. Oxidative stress is commonly associated with mitochondrial dysfunction, and vice versa, mitochondrial dysfunction causes ROS overproduction and development of oxidative stress [35]. So we further investigated the effect of curcumin on the mitochondrial ROS. Treatment with curcumin in THP-1 and RAW264.7 cells largely inhibited the increase of mitochondrial ROS induced by MSU crystal stimuli. To some extent, these data reflect that curcumin may inhibit the activity of NLRP3 inflammasome caused by MSU crystal stimuli through preventing mitochondrial damage.

To more accurately evaluate the effect of curcumin on MSU crystal-induced inflammation, we established two types of mouse models of gouty arthritis by injecting MSU crystals into footpads and ankle joints. The process induced a series of inflammatory responses, similar to those that occur in acute gouty arthritis [36]. In MSU-injected footpads and ankle joint, curcumin treatment alleviated the swelling of the paw and ankle joints, and the MPO and HE assays showed that curcumin relieved the recruitment of neutrophils in the footpads. Curcumin administration also inhibited NF-κB activation, and the expression of TLR4, MyD88, and NLRP3 in the mouse model of gout arthritis. However, gout is not only related to high uric acid levels, but also genetic and environmental factors. Some patients have high levels of uric acid over a long period of time and do not develop gout. The gout mouse model we used was triggered only by MSU crystals, so further clinical trials are needed to determine whether the use of curcumin should be recommended in gout patients.

## Conclusions

Our study indicates that curcumin plays an anti-inflammatory role in MSU-induced inflammation by suppressing the degradation of IκBα, the NF-κB signaling pathway, the NLRP3 inflammasome activation, and the mitochondrial damage, strongly suggesting that curcumin may be used as a potential drug for the treatment of gout in the future.

## Data Availability

The datasets used and/or analyzed during the present study are available from the corresponding author on reasonable request.

## Abbreviations

Cur: Curcumin
MMP: Mitochondrial membrane potential
MPO: Myeloperoxidase
MSU: Monosodium urate
NALP3: NLR family pyrin domain containing 3
PGE2: Prostaglandin E2
PMA: Phorbol myristate acetate
ROS: Reactive oxygen species
TLR: Toll-like receptor
Veh: Vehicle
